# Relationship between Insulin Resistance and Coronary Artery Calcium in Young Men and Women

**DOI:** 10.1371/journal.pone.0053316

**Published:** 2013-01-16

**Authors:** Ki-Chul Sung, Jin-Ho Choi, Hyeon-Cheol Gwon, Seung-Hyuk Choi, Bum-Soo Kim, Hyon Joo Kwag, Sun H. Kim

**Affiliations:** 1 Division of Cardiology, Department of Medicine, Kangbuk Samsung Hospital, Sungkyunkwan University School of Medicine, Seoul, Republic of Korea; 2 Division of Cardiology, Cardiac and Vascular Center, Department of Medicine, Samsung Medical Center, Sungkyunkwan University School of Medicine, Seoul, Republic of Korea; 3 Department of Radiology, Kangbuk Samsung Hospital Sungkyunkwan, University School of Medicine, Seoul, Republic of Korea; 4 Division of Endocrinology, Department of Medicine, Stanford University School of Medicine, Stanford, California, United States of America; Indiana University School of Medicine, United States of America

## Abstract

**Background:**

The gender disparity in cardiovascular disease (CVD) risk is greatest between young men and women. However, the causes of that are not fully understood. The objective of this study was to evaluate the relationship between insulin resistance and the presence of coronary artery calcium (CAC) to identify risk factors that may predispose young men and women to CVD.

**Methodology/Principal Findings:**

Insulin resistance and CVD risk factors were examined in 8682 Korean men and 1829 women aged 30–45 years old. Insulin resistance was estimated using the homeostasis model assessment of insulin resistance (HOMA-IR), and CAC was measured using computed tomography. Women were less likely to be insulin resistant (upper quartile of HOMA-IR, 18% vs. 27%, p<0.001) and had a lower prevalence of CAC (1.6% vs. 6.4%, p<0.001). Even when equally insulin resistant men and women were compared, women continued to have lower prevalence of CAC (3.1% vs. 7.2%, p = 0.004) and a more favorable CVD risk profile. Finally, after adjustment for traditional CVD risk factors, insulin resistance remained an independent predictor of CAC only in men (p = 0.03).

**Conclusions/Significance:**

Young women have a lower risk for CVD and a lower CAC prevalence compared with men. This favorable CVD risk profile in women appears to occur regardless of insulin sensitivity. Unlike men, insulin resistance was not a predictor of CAC in women in this cohort. Therefore, insulin resistance has less impact on CVD risk and CAC in young women compared with men, and insulin resistance alone does not explain the gender disparity in CVD risk that is observed at an early age.

## Introduction

Women have a lower risk for cardiovascular disease (CVD) than men of equal age [1], [2]. This disparity in CVD risk narrows with aging [1], [3] and the presence of diabetes [4], [5], [6]. Both aging and diabetes are associated with increased prevalence of insulin resistance and insulin-resistance related CVD risk factors, including dysglycemia, dyslipidemia, and hypertension [7], [8]. These risk factors are present less commonly in young women compared with similarly aged men, despite the increased adiposity that is observed in women [9]. Therefore, one simple explanation for the gender disparity in CVD risk factors has been that young women are more insulin sensitive than young men [10], [11]. Another explanation could be that, for a given level of insulin resistance, young women may have fewer CVD risk factors.

To examine these two possibilities, we evaluated the relationship between insulin sensitivity and CVD risk factors, including presence of coronary artery calcium (CAC), in 8682 Korean men and 1829 women aged 30–45 years old. To the best of our knowledge, this study contains the largest population of young adults characterized by measurements of CAC and other CVD risk factors. In addition, this is the first study to evaluate the role of insulin resistance in modulating gender disparities in CAC in young adults.

## Methods

### Subjects

The study population consisted of patients aged 30–45 years old who participated in a comprehensive health examination in 2010 at Kangbuk Samsung Hospital, College of Medicine, Sungkyunkwan University. Initially, 10596 individuals were identified who met the age criterion. Individuals were excluded for the following reasons: missing weight (n = 10), unclear diabetes status (n = 47), unclear coronary disease history (n = 2), and reported history of coronary artery disease (n = 26). After exclusion, 8682 men and 1829 women were included.

The study was approved by the institutional review board at Kangbuk Samsung Hospital. Informed consent requirement was waived because personal identifying information was not accessed.

### Data Collection

The health examination included a medical history, physical examination, fasting blood samples and an imaging study for assessment of CAC. Trained clinical staff measured weight, height and blood pressure. Body mass index (BMI) was calculated by dividing weight (kilogram) by height (meters) squared.

Patients also completed self-administered questionnaires related to their medical and social histories. Individuals were asked to designate their highest level of education. They were classified as having higher education if they had completed 16 or more years of school. Smoking status was reported as never, past, or current. For the current study, only current smoking status was considered. Hypertension was diagnosed if individuals met one of the following criteria: systolic blood pressure ≥140 or diastolic blood pressure ≥90 mmHg [12], history of hypertension, or use of anti-hypertensive medications. Diabetes was diagnosed when individuals had a fasting glucose concentration ≥126 mg/dL [13], a prior history of diabetes, or treatment with anti-diabetic medications. Type of diabetes was not differentiated in this study.

Blood samples were collected after an overnight fast. Fasting plasma glucose and lipid profile were measured using Bayer Reagent Packs on an automated chemistry analyzer (Advia 1650 Autoanalyzer; Bayer Diagnostics, Leverkusen, Germany). Insulin concentration was measured with the electrochemiluminescence immunoassay (Roche Diagnostics, Mannheim, Germany) with a repeatability and precision coefficient of variation of 0.8–1.5% and 2.4–4.9%, respectively. High-sensitivity C-reactive protein levels were measured using a nephelometric assay (BNII nephelometer, Dade Behring, Deerfield, IL). The limit of measurement was 1.67 nmol/L with a sample dilution of 1∶20.

To measure CAC, a 64-slice multidetector computed tomography scanner (Lightspeed VCT XTe-64 slice; GE Healthcare, Milwaukee,WI) was used. A standard scanning protocol was employed: 32×0.625-mm section collimation, 400-msec rotation time, 120-kV tube voltage, and 31 mAS (310 mA*0.1 sec) tube current under electrocardiographic-gated dose modulation. The Agatston scoring method was used to quantify CAC [14]. CAC scores were positively skewed with 95% having zero value. Therefore, coronary calcification was defined as the presence of any calcium (CAC>0).

### Calculations

The homeostasis model assessment of insulin resistance (HOMA-IR) was calculated using fasting plasma glucose and insulin concentration: [fasting glucose (mmol/L) X fasting insulin (mU/L)/22.5] [15]. Framingham risk score was also calculated using gender-specific equations [16].

### Statistical Analysis

Continuous variables were expressed as mean ± SD or median [interquartile range] if not normally distributed. Continuous variables were compared using the independent t-test. Nonparametric variables were log-transformed prior to analyses. Categorical variables were expressed as percentage and compared using the chi-squared test.

To better understand the role of insulin resistance on gender differences in CVD risk, men and women were classified based on HOMA-IR as insulin sensitive (lowest quartile of HOMA-IR) or insulin resistant (highest quartile of HOMA-IR). Individuals with diabetes were separately evaluated, as previous studies have shown differential CVD risk in individuals with diabetes compared with those without diabetes. Differential CVD risk was especially apparent in women [5], [6]. CVD risk factors and CAC>0 were then compared between men and women matched for insulin sensitivity or diabetes status. Crude and adjusted logistic regression analyses also were used to determine the association between CAC>0 and insulin resistance or diabetes. Covariates in the model included traditional risk factors for CVD: age, current smoking status (yes, no), hypertension (yes, no), low density lipoprotein cholesterol (LDL-C) and high density lipoprotein cholesterol (HDL-C) concentration. In a secondary model, BMI was also included as a covariate in addition to the traditional CVD risk factors. P≤0.05 was considered significant. All statistical analysis was conducted using SPSS (version 16 for Windows; SPSS, Chicago, IL).

## Results

Characteristics of the young men and women are shown in Table 1. Despite similar age, most CVD risk factors were more favorable in women. Women also had a lower HOMA-IR and a lower prevalence of diabetes. Given the more favorable CVD risk profile, women had a lower Framingham risk score and a lower prevalence of CAC.

**Table 1:** Characteristics of the study population by gender.

	Men (n = 8682)	Women (n = 1829)	p Value
Age, years	38.8±4.2	38.9±4.1	0.19
BMI, kg/m^2^	25.0±3.0	22.4±3.4	<0.001
Higher education, no. (%)	6685 (80%)	1106 (62%)	<0.001
Current smoker, no. (%)	2595 (30%)	23 (1%)	<0.001
Diabetes, no. (%)	332 (3.8%)	37 (2%)	<0.001
Blood pressure, mmHg			
Systolic blood pressure	119±11	108±12	<0.001
Diastolic blood pressure	76±9	68±9	<0.001
Lipids, mmol/L			
LDL-C	3.3±0.8	2.9±0.8	<0.001
HDL-C	1.3±0.3	1.6±0.4	<0.001
Triglyceride	1.4	0.9	<0.001
	[1.0,2.0]	[0.7,1.2]	
Glucose, mmol/L	5.3±0.8	5.1±0.7	<0.001
Hemoglobin A1c, %	5.67±0.53	5.64±0.43	0.02
Insulin, pmol/L	38	33	<0.001
	[25,54]	[22,47]	
HOMA-IR	1.26	1.05	<0.001
	[0.83, 1.88]	[0.70, 1.54]	
High-sensitivity C-reactive protein,	5.7	3.8	<0.001
nmol/L	[2.9,10.5]	[2.9,7.6]	
Framingham 10 year risk, %	4	1	<0.001
	[3,6]	[1,2]	
CAC>0, no. %	553 (6.4%)	29 (1.6%)	<0.001
CAC>100, no. %	56 (0.6%)	2 (0.1%)	0.003

Data are mean ± SD or median [interquartile range] unless otherwise noted. LDL-C, low density lipoprotein cholesterol; HDL-C, high density lipoprotein cholesterol; CAC, coronary artery calcium.

To better understand the role of insulin resistance and diabetes on gender differences, we compared CVD risk factors between men and women with similar states of insulin sensitivity, insulin resistance or overt diabetes (Table 2). Beginning with the insulin sensitive group, more women qualified as being insulin sensitive compared with men (33% vs. 23%, p<0.001). Despite having similar age and HOMA-IR in insulin-sensitive men and women, women had a more favorable CVD risk profile, with the exception of HgA1c, which was slightly higher in women despite having a lower fasting glucose.

**Table 2:** Cardiovascular risk factors in young men and women by insulin resistance and diabetes status.

	No Diabetes						Diabetes		
	Insulin Sensitive			Insulin Resistant			–		
	(HOMA-IR <0.79)			(HOMA-IR ≥1.76)					
	Men	Women	p	Men	Women	p	Men	Women	p
	(n = 1950)	(n = 585)	Value	(n = 2213)	(n = 323)	Value	(n = 332)	(n = 37)	Value
HOMA-IR	0.6	0.6	0.09	2.3	2.3	0.96	2.6	2.9	0.82
	[0.4,0.7]	[0.4,0.7]		[2.0,2.9]	[2.0,2.9]		[1.6, 3.9]	[1.4,3.9]	
Age, years	38.9±4.2	38.8±4.3	0.49	38.5±4.1	39.5±3.9	<0.001	41.4±2.7	41.2±3.4	0.61
BMI, kg/m^2^	23.1±2.4	21.0±2.4	<0.001	27.0±3.0	25.3±4.2	<0.001	26.6±3.3	25.7±5.1	0.16
Higher education, no. (%)	1499	376	<0.001	1676	166	<0.001	225	16	0.003
	(79%)	(66%)		(79%)	(53%)		(69%)	(43%)	
Current smoker, no. (%)	587	10	<0.001	682	5	<0.001	135	0	<0.001
	(30%)	(2%)		(31%)	(2%)		(41%)	(0%)	
Blood pressure, mmHg									
Systolic blood pressure	115±11	105±11	<0.001	122±12	113±12	<0.001	122±13	113±12	<0.001
Diastolic blood pressure	73±8	66±8	<0.001	78±9	71±9	<0.001	78±9	72±8	<0.001
Lipids, mmol/L									
LDL-C	3.2±0.8	2.8±0.8	<0.001	3.4±0.8	3.2±0.8	<0.001	3.2±1.0	3.3±0.9	0.58
HDL-C	1.5±0.3	1.7±0.3	<0.001	1.2±0.2	1.4±0.3	<0.001	1.2±0.3	1.4±0.3	<0.001
Triglyceride	1.0	0.7	<0.001	1.9	1.3	<0.001	1.9	1.6	0.002
	[0.7,1.3]	[0.6,0.9]		[1.4, 2.6]	[1.0,1.8]		[1.3,2.8]	[0.9,2.4]	
Glucose, mmol/L	4.9±0.4	4.7±0.4	<0.001	5.5±0.5	5.4±0.5	<0.001	8.0±2.4	7.9±2.8	0.83
Insulin, pmol/L	18	18	0.35	67	68	0.43	54	60	0.66
	[14,21]	[14,22]		[58,82]	[59,84]		[35,79]	[31,72]	
HgA1c, %	5.5±0.2	5.6±0.3	0.01	5.7±0.3	5.7±0.3	0.12	7.4±1.6	7.3±1.7	0.87
Hs-CRP, nmol/L	3.8	2.9	<0.001	7.6	6.7	0.008	8.6	7.6	0.39
	[2.9,8.6]	[2.9,4.8]		[4.8,14.3]	[3.8, 12]		[4.8,17]	[3.8,14]	
Framingham 10 year risk	3	1	<0.001	5	1	<0.001	7	4	<0.001
(%)	[1,6]	[1,1]		[1,8]	[1,1]		[6,11]	[3,6]	

Data are mean ± SD or median [interquartile range] unless otherwise noted. LDL-C, low density lipoprotein cholesterol; HDL-C, high density lipoprotein cholesterol.

In the insulin resistant group, the HOMA-IR was more than three times higher than that in the insulin sensitive group. There were more men than women (27% vs.18%, p<0.001) in the insulin resistant group, but HOMA-IR was similar in men and women. In both men and women, values of CVD risk factors were worse in the insulin resistant group compared with the insulin sensitive group. However, within the insulin resistant group, women again maintained a more favorable CVD risk profile.

The prevalence of diabetes was low in this young cohort. Men and women with diabetes were more similar in age, BMI, and glucose indices compared with men and women in the other groups, although men had a higher prevalence of diabetes compared with women (3.8% vs. 2%, p<0.001). Despite being more comparable in demographic and metabolic variables, women still had significantly lower blood pressure and triglyceride concentration and higher HDL-C concentration than men. As a result, Framingham risk score was also significantly lower in women compared with men.

**Figure 1:**
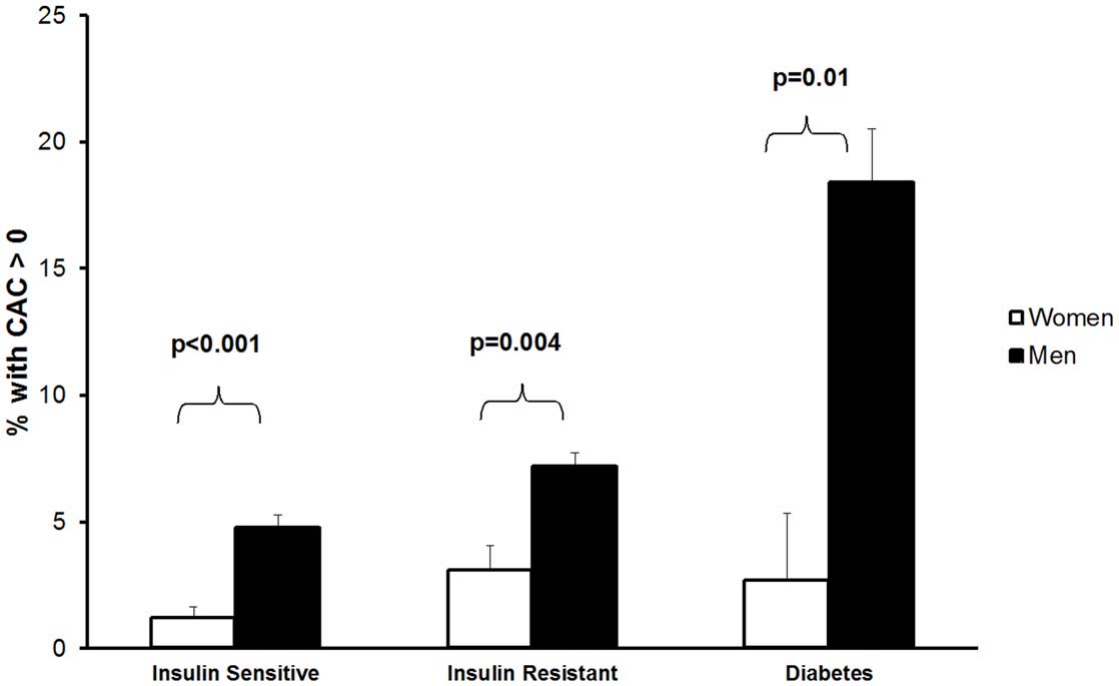
Proportion with detectable CAC by insulin resistance and diabetes status in young women and men. Regardless of the classification, men were significantly more likely to have CAC. P refers to difference in proportion between genders. Error bars represent standard error.

Figure 1 shows the prevalence of CAC stratified by insulin resistance and diabetes status in men and women. Regardless of the category, the proportion of individuals with CAC was greater in men than women. Nonetheless, in both men and women, the proportion with CAC increased significantly in insulin resistant individuals compared with insulin sensitive individuals. For men, there was a 1.5 fold increase in prevalence of CAC in insulin resistant individuals compared with insulin sensitive individuals (p = 0.001); for women, there was a 2.6 fold increase in prevalence of CAC in insulin resistant individuals compared with insulin sensitive individuals, but this difference did not reach statistical significance (p = 0.07).

For men, the proportion with CAC was highest in those with diabetes. As high as 18% of the men with diabetes had detectable CAC, which was 3.8 fold greater than men classified as insulin sensitive without diabetes (p<0.001). In contrast, for women, the proportion of those with diabetes with CAC was not statistically different from those who were insulin sensitive or insulin resistant with CAC (p≥0.39).

Table 3 shows the association between CAC and insulin resistance and diabetes. For men, HOMA-IR and diabetes were significantly associated with the presence of CAC, even when adjusted for traditional CVD risk factors. When this association was further adjusted for BMI, HOMA-IR and the presence of diabetes remained significantly associated with CAC. For women, insulin resistance, defined as being in the upper quartile of HOMA-IR, was significantly associated with CAC. However, this association was no longer significant when adjusted for CVD risk factors. For women, diabetes was not significantly associated with CAC.

**Table 3:** Odds ratio (OR) for coronary artery calcium by insulin resistance and diabetes status in young men and women.

	Univariate			Multivariate Model 1*			Multivariate Model 2**		
Men	OR	95% CI	p	OR	95% CI	p	OR	95% CI	p
HOMA-IR, log	**1.50**	**1.31, 1.73**	**<0.001**	**1.22**	**1.05, 1.41**	**0.01**	**1.19**	**1.01, 1.41**	**0.04**
Upper Quartile of HOMA-IR (yes, no)	**1.57**	**1.31, 1.87**	**<0.001**	**1.25**	**1.03, 1.52**	**0.03**	1.20	0.97, 1.48	0.09
Diabetes (yes, no)	**3.60**	**2.68, 4.82**	**<0.001**	**2.23**	**1.64, 3.04**	**<0.001**	**2.19**	**1.61, 2.98**	**<0.001**
**Women**									
HOMA-IR, log	1.48	0.84, 2.62	0.18	0.86	0.47, 1.58	0.63	0.83	0.43, 1.61	0.59
Upper Quartile of HOMA-IR (yes, no)	**2.28**	**1.05, 4.94**	**0.04**	1.22	0.51, 2.89	0.66	1.25	0.49, 3.17	0.65
Diabetes (yes, no)	1.75	0.23, 13.2	0.59	0.79	0.10, 6.31	0.83	0.78	0.10, 6.42	0.82

* Model 1 is adjusted for age, smoking status, hypertension, LDL-C, HDL-C;

** Model 2 is adjusted for variables in Model 1 and BMI.

## Discussion

Although both young men and women are at low risk for CVD [17], we found a clear gender disparity in risk for CVD in our cohort. Overall, young women had a better CVD risk profile and lower CAC compared with men. Women were also more likely to be insulin sensitive which might have explained the gender disparity in CVD risk. On the other hand, even when matched for level of insulin resistance, young women maintained a lower CVD risk profile and prevalence of CAC. Therefore, women had a better CVD risk profile independent of insulin sensitivity.

To the best of our knowledge, our study contains the largest cohort of young adults (aged 45 and less) characterized by measurement of CAC. In previous studies of young adults with CAC, the population sample has ranged from 630 to 3043 individuals [3], [18], [19], [20]. In those studies, the prevalence of CAC has ranged from 11–31% in men and 4–10% in women, with men having 2–4 times greater prevalence of CAC compared with women [3], [18], [19], [20]. Similarly, we show here that men have approximately a four-fold increase in CAC compared with women. The lower overall prevalence of CAC in our study may reflect differences in race [21], [22] and age.

Our study is also unique because it evaluates the role of insulin resistance in mediating the gender disparity in CAC in young adults. Previous studies have suggested that women without diabetes may be more insulin sensitive compared with men [10], [23], [24], which could drive the development of CAC and CVD over time. However, those studies included individuals with wider age ranges than the current study and did not match men and women based on insulin resistance. In our study, when men and women were specifically matched for level of insulin resistance, women continued to have a more favorable CVD risk profile and lower CAC compared with men, suggesting that insulin resistance does not solely mediate the risk difference between young women and men.

Insulin resistance was also not an independent predictor of CAC in women as it is in men. In older cohorts, studies have shown that insulin resistance has a significant impact on CAC in both men and women [25], [26]. In our study, insulin resistance was not a significant predictor of CAC when adjusted for other CVD risk factors. This finding may relate to the low prevalence of CAC in women in this cohort; a greater number of women may be required to observe a measurable effect of insulin resistance on CAC. Our results also suggest that the immediate clinical impact of insulin resistance may be minimal in young women given their low overall risk. As seen in Table 2, CVD risk factors were extremely favorable in women classified as being insulin sensitive. Although the absolute values of CVD risk factors worsened in women with insulin resistance, they remained normal according to accepted criteria for CVD risk [27]. For example, the median triglyceride concentration in insulin resistant women was 1.3 mmol/L, which is almost twice the value of the triglyceride concentration in insulin sensitive women (0.7 mmol/L). However, a triglyceride concentration of 1.3 mmol/L does not meet the cut-point for CVD risk of 1.7 mmol/L [27]. In comparison, insulin resistant men also had a near doubling of triglyceride concentration compared with insulin sensitive men. However, in contrast to women, insulin resistant men had a median triglyceride concentration of 1.9 which is above the risk cut-point. Therefore, although insulin resistance was associated with a less favorable CVD risk profile in both women and men, the incremental impact of insulin resistance on CVD risk factors and thus CAC was lower in young women compared with men due to the lower baseline risk in women.

The lack of impact of diabetes status on CAC in women deserves mention. In older cohorts, diabetes has been suggested to increase CAC prevalence and progression in both men and women [28], [29]. The impact of diabetes on atherosclerotic risk is not isolated to individuals with type 2 diabetes and has been observed in women with type 1 diabetes [30]. Therefore, factors beyond insulin resistance may account for the higher CVD risk in individuals with diabetes. In our study, men with diabetes had a significant 3.8-fold increase in CAC prevalence compared with insulin sensitive men without diabetes. In contrast, women with diabetes had a 2.5-fold increase in CAC prevalence, but this was not statistically significant. The lack of a statistical effect may relate to the small sample of women with diabetes in this cohort. In addition, some of the differential impact of diabetes by gender may relate to the observation that men with diabetes had greater CVD risk factors compared with men without diabetes (e.g., greater smoking). Finally, the immediate impact of diabetes on CVD risk may be low in young women because of their low baseline risk.

Compared with older cohorts, the majority of individuals with CAC had low calcium scores (<100), which were lower than the threshold that were previously shown to predict future coronary artery disease [31], [32]. Therefore, the significance of CAC in this young cohort could be debated. On the other hand, it is remarkable that insulin resistance and diabetes were significantly associated with CAC in men, even at this young age. In addition, since baseline presence of CAC is a strong predictor of rate of progression of coronary calcification [33], individuals with early CAC are likely to be at the highest risk for future coronary artery disease compared with their cohorts without CAC.

There are several limitations of this study. First, we had more men than women. However, our cohort had more women than in previous studies that have measured CAC in young cohorts [3], [18], [19], [20]. Second, men in our cohort smoked more than women, which could partially explain the disparity in CAC prevalence between genders. However, the prevalence of current smokers was similar between insulin sensitive and resistant groups within genders; therefore, smoking status is unlikely to be responsible for the observed differences between insulin sensitive and resistant groups. Third, we did not ascertain a family history of premature coronary artery disease in our cohort, which could have increased the risk for CAC in this young adult population. Fourth, we used HOMA-IR as a surrogate measure of insulin resistance. Although significantly associated with direct measures of insulin resistance [34], HOMA-IR might have misclassified insulin sensitivity status. Finally, our study was a cross-sectional study and thus provides a snapshot of the association between insulin resistance and CAC, which is a reflection of calcified plaque burden in coronary arteries. Therefore, we cannot discount the impact of insulin resistance on future CVD in women and on noncalcified atherosclerosis.

In conclusion, young women have a lower risk for CVD and a lower CAC prevalence compared with men. This favorable CVD risk profile in women appears to occur regardless of insulin sensitivity. In addition, unlike men, insulin resistance was not a predictor of CAC in women. Therefore, insulin resistance has less impact on CVD risk and CAC in young women compared with men, and insulin resistance alone does not explain the gender disparity in CVD risk that is observed at an early age.
